# Amelogenic transcriptome profiling in ameloblast-like cells derived from adult gingival epithelial cells

**DOI:** 10.1038/s41598-019-40091-x

**Published:** 2019-03-06

**Authors:** Sun-Yi Hyun, Seyoung Mun, Kyung-Jung Kang, Jong-Chan Lim, Shin-Young Kim, Kyudong Han, Young-Joo Jang

**Affiliations:** 1 Department of Nanobiomedical Science and BK21 PLUS Global Research Center for Regenerative Medicine, Dankook University, Cheonan, 31116 South Korea; 2 DKU-Theragen institute for NGS analysis (DTiNa), Cheonan, 31116 South Korea

## Abstract

Dental enamel is the highly mineralized tissue covering the tooth surface and is formed by ameloblasts. Ameloblasts have been known to be impossible to detect in adult tooth because they are shed by apoptosis during enamel maturation and tooth eruption. Owing to these, little was known about appropriate cell surface markers to isolate ameloblast-like cells in tissues. To overcome these problems, epithelial cells were selectively cultivated from the gingival tissues and used as a stem cell source for ameloblastic differentiation. When gingival epithelial cells were treated with a specified concentration of BMP2, BMP4, and TGFβ-1, the expression of ameloblast-specific markers was increased, and both the MAPK and Smad signaling pathways were activated. Gingival epithelial cells differentiated into ameloblast-like cells through epithelial-mesenchymal transition. By RNA-Seq analysis, we reported 20 ameloblast-specific genes associated with cell surface, cell adhesion, and extracellular matrix function. These cell surface markers might be useful for the detection and isolation of ameloblast-like cells from dental tissues.

## Introduction

Dentin, dental pulp, periodontal ligament, and dental enamel are developed by reciprocal interactions between dental epithelium and ectomesenchyme. Neural crest cell-derived ectomesenchyme differentiates into odontoblasts, periodontal ligament progenitors, cementoblasts, as well as various fibroblasts. On the other hand, enamel-forming ameloblasts differentiate from epithelial cells originating from oral ectoderm. In the process of enamel formation, the inner enamel epithelium differentiates into ameloblasts^1^. Ameloblastic differentiation possibly occurs after the initial dentin matrix protein secretion and deposition by odontoblasts^2,3^. The enamel matrix proteins (EMPs) are degraded by various proteinases secreted by ameloblasts and replaced by minerals during the maturation stage^4^. Hertwig’s epithelial root sheath/epithelial cell rests of Malassez (HERS/ERM) have been reported to be a unique epithelial cell source^5,6^. Bone marrow stromal cells, embryonic stem cells, and skin epithelial cells are alternative sources for the construction of ameloblasts^7^. Induction mechanism of various progenitors is strictly regulated by growth factors and cytokines, such as TGFs, FGFs, Wnts, and BMPs, as well as the extracellular matrix in the epithelium and mesenchyme^8,9^. In ameloblastic differentiation, BMP2 and BMP4 are secreted by ectomesenchymal odontoblasts and play important roles in the expression of EMPs and terminal differentiation of ameloblasts^10,11^. Ameloblast differentiation is prevented by follistatin by antagonizing the inductive effect of BMP4 from the odontoblasts. The expression of follistatin is shown to be induced by activin A from the overlying mesenchymal follicle cells. Thus, a balance between BMP4 and activin A, is required for proper ameloblast differentiation^12^. In addition, knockout of a BMP receptor, Bmpr1a/ALK3, causes defective enamel formation on tooth crowns^13^. Besides BMPs, TGFβ-1 stimulates the expression and secretion of EMPs in ameloblasts. The inhibition of the TGFβ-1 signaling pathway causes tooth and enamel malformations^14,15^. The Smad signaling is known as an intracellular canonical pathway activated by TGF-β superfamily members through a heteromeric receptor complex, comprised of type I and type II receptors^16,17^. According to the activation of receptors by TGFβ-1 and BMPs, Smad2/3 and Smad1/5/8, which are known as the regulatory Smads (R-Smads) are phosphorylated, respectively, and then, a complex of phospho-R-Smads and Smad4 regulates the expression of target genes in the nucleus^18,19^.

In this study, we isolated and characterized the epithelial cells from human gingival tissue, which is comparatively easy to obtain, and successfully induced differentiation into ameloblast-like cells through epithelial-mesenchymal transition. In addition, we revealed potential surface markers of ameloblast-like cells, which are categorized into those involved in cell adhesion and extracellular matrix functions.

## Results

### Culture of the epithelial cells derived from human gingival tissue

**Figure 1:**
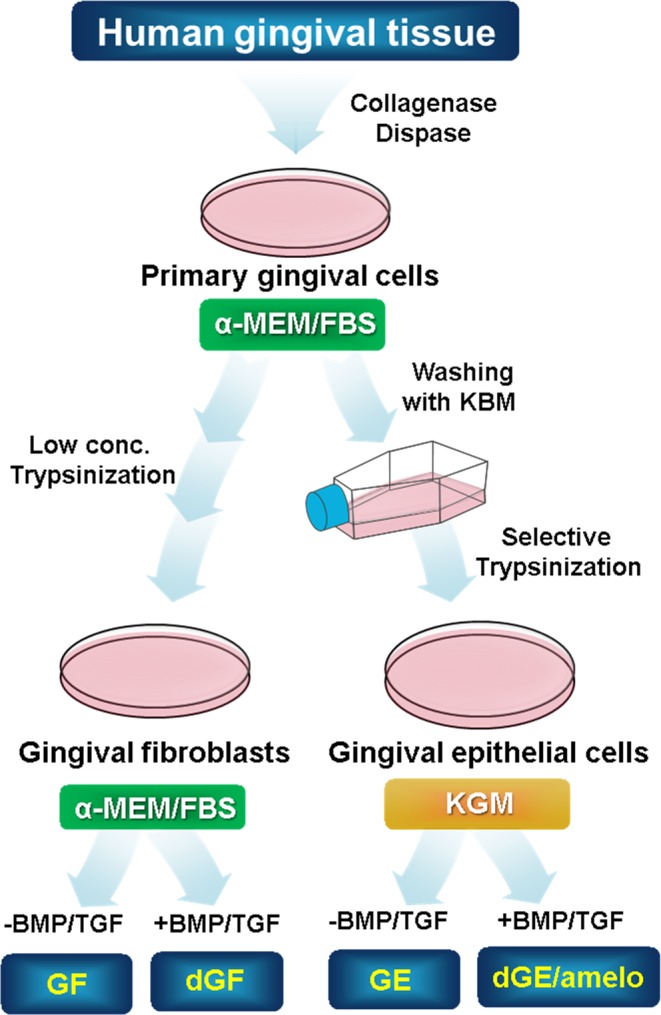
Schematic strategy of the epithelial cell culture derived from human gingival tissue. The detailed scheme of the selective cell culture process was described in Materials and Methods.

**Figure 2:**
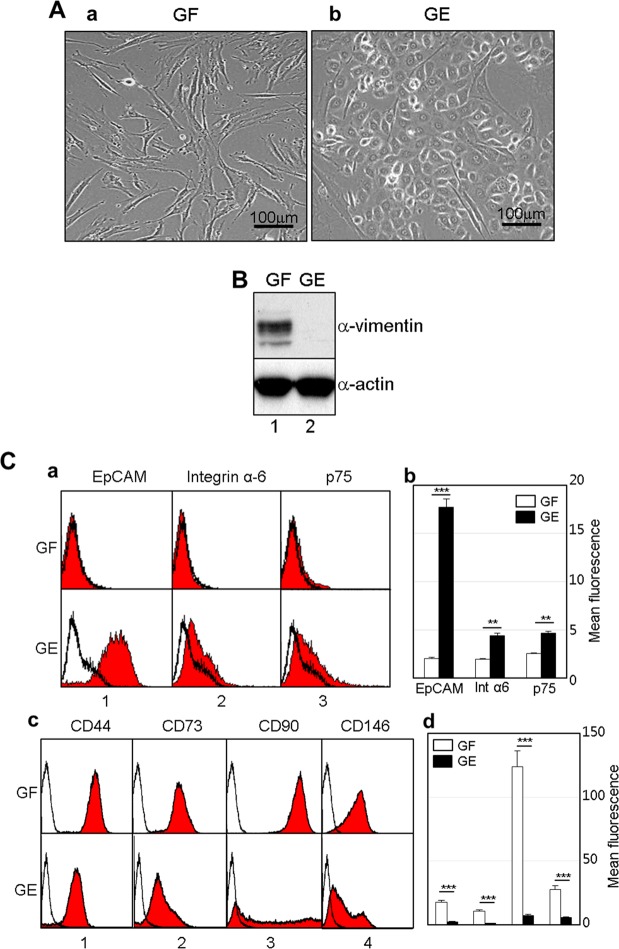
Characterization of the gingival epithelial cells cultured from human gingival tissue. (**A**) Microscopic observation of cellular morphology of the gingival fibroblasts (GF) (*a*) and the gingival epithelial cells (GE) (*b*). (**B**) Expression of endogenous vimentin, a fibroblastic cell marker in GF and GE. Total cell extracts were analyzed on SDS-PAGE, and endogenous vimentin was detected by western blot analysis with anti-vimentin antibody (*α-vimentin*). For normalization of protein amounts used, actin was detected by anti-actin antibody (*α-actin*). (**C**) Immunophenotyping of cells by using epithelial and mesenchymal cell markers. Intact cells harvested by non-enzymatic method were incubated with the primary antibodies, and expression of the cell surface antigens was analyzed by FACS as described in Materials and Methods. The mean fluorescence values from the FACS data were estimated from peak data using Cell Quest software and the WinMDI 2.9 program. ***a & b***, FACS histogram and mean fluorescence value on the expression of epithelial cell markers. ***b & d***, FACS histogram and mean fluorescence value on the expression of mesenchymal cell markers. The data were originated from a cell line of 4 individual cultures of gingival fibroblast (**A**, **B**, a & c in **C**), and from average value of 4 individual cultures (b & d in C). *P < 0.1, **P < 0.01, or ***P < 0.001 was determined by using Student t-test.

To establish ameloblast-like cells from commonly available dental tissue, we at first attempted to isolate the epithelial cells from gingival tissue of ten donors (Fig. 1). Fibroblastic cells mostly grew out from gingival tissue under continuous culture in α-MEM/20% FBS. However, gingival epithelial cells were obtained within 1–2 weeks through selective transfer culture in a serum-free keratinocyte growth medium. During selective culture, residual fibroblastic cells were selectively eliminated by treatment with a low concentration of trypsin. The gingival fibroblasts exhibited bipolar fibroblastic shapes, whereas the gingival epithelial cells exhibited polygonal shapes that are a typical cellular morphology of epithelial cells (Fig. 2A). The expression of vimentin, a typical fibroblast marker, dramatically decreased in epithelial cells (Fig. 2B). Integrin α-6, EpCAM, and p75NTR have been used as epithelial stem cell markers in human HERS/ERM and ectomesenchymal stem cells^20,21^. The expressions of EpCAM, integrin α-6, and p75NTR were 8.9, 2.3, and 1.9 times greater in gingival epithelial cells than in gingival fibroblasts, respectively (Fig. 2C, a & b). On the other hand, the expressions of CD44, CD73, CD90, and CD146, which are known as mesenchymal stem cell markers^22–25^, in gingival epithelial cells were 5.5, 8.0, 16.7, and 3.9 times lower than those in fibroblasts, respectively (Fig. 2C, c & d). These results indicated that although most of the primary cells cultured from adult gingival tissue grew as fibroblasts, epithelial cells present in small amounts can be grown using selective trypsinization and specific medium.

### Co-treatment with BMPs and TGFβ-1 induces ameloblastic differentiation of gingival epithelial cells

**Figure 3:**
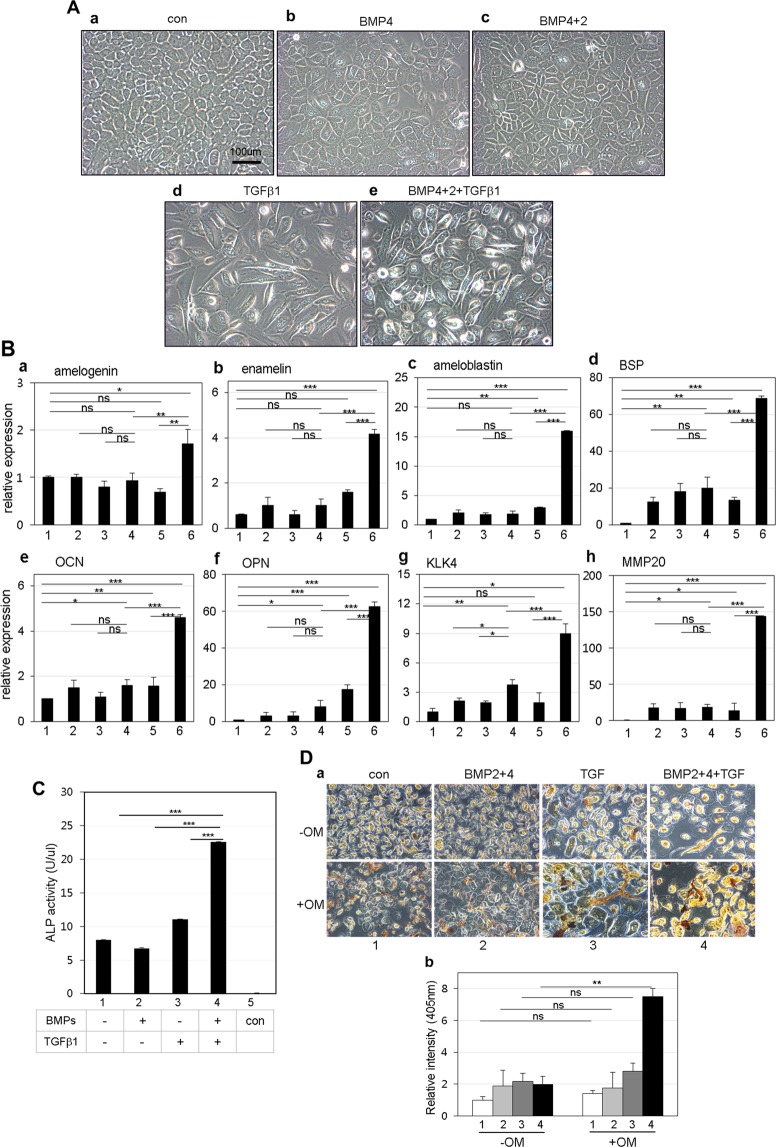
Co-treatment of BMP2, BMP4, and TGFβ-1 induces ameloblastic cytodifferentiation in the gingival epithelial cells. (**A**) Microscopic observation of cellular morphology of cells treated with cytokines for 7 days. *a*, cells without treatment; *b*, cells treated with BMP4; *c*, cells co-treated with BMP4 and BMP2; *d*, cells treated with TGFβ-1; *e*, cells co-treated with BMP4, BMP2, and TGFβ-1. (**B**) Relative mRNA expressions of amelogenic and osteogenic markers in cells treated with cytokines. mRNA expression was analyzed by qPCR as described in the Materials and Methods. See also Table S1. *a*, expression of amelogenin; *b*, expression of enamelin; *c*, expression of ameloblastin; *d*, expression of bone sialoprotein; *e*, expression of osteocalcin; *f*, expression of osteopontin; *g*, expression of KLK4; *h*, expression of MMP20. 1, control without treatment; 2, treatment with BMP2; 3, treatment with BMP4; 4, co-treatment with BMP4 and BMP2; 5, treatment with TGFβ-1; 6, co-treatment with BMP4, BMP2, and TGFβ-1. The data were originated from average value of 3 individual cultures. Statistical significance of *P < 0.1, **P < 0.01, or ***P < 0.001 was determined by using Student t-test. (**C**,**D**) Estimation of alkaline phosphatase activity and mineralization efficiency. Epithelial cells were incubated in KGM containing 50 μg/ml ascorbic acid, 10 mM β-glycerophosphate, and 5 μM dexamethasone for 7~14 days after ameloblastic cytodifferentiation. The data were originated from average value of 2 individual cultures. Statistical significance of *P < 0.1, **P < 0.01, or ***P < 0.001 was determined by using Student t-test. 1, without treatment; 2, co-treatment with BMP4 and BMP2; 3, treatment with TGFβ-1; 4, co-treatment with BMP4, BMP2 & TGFβ-1; 5, control of buffer only.

**Figure 4:**
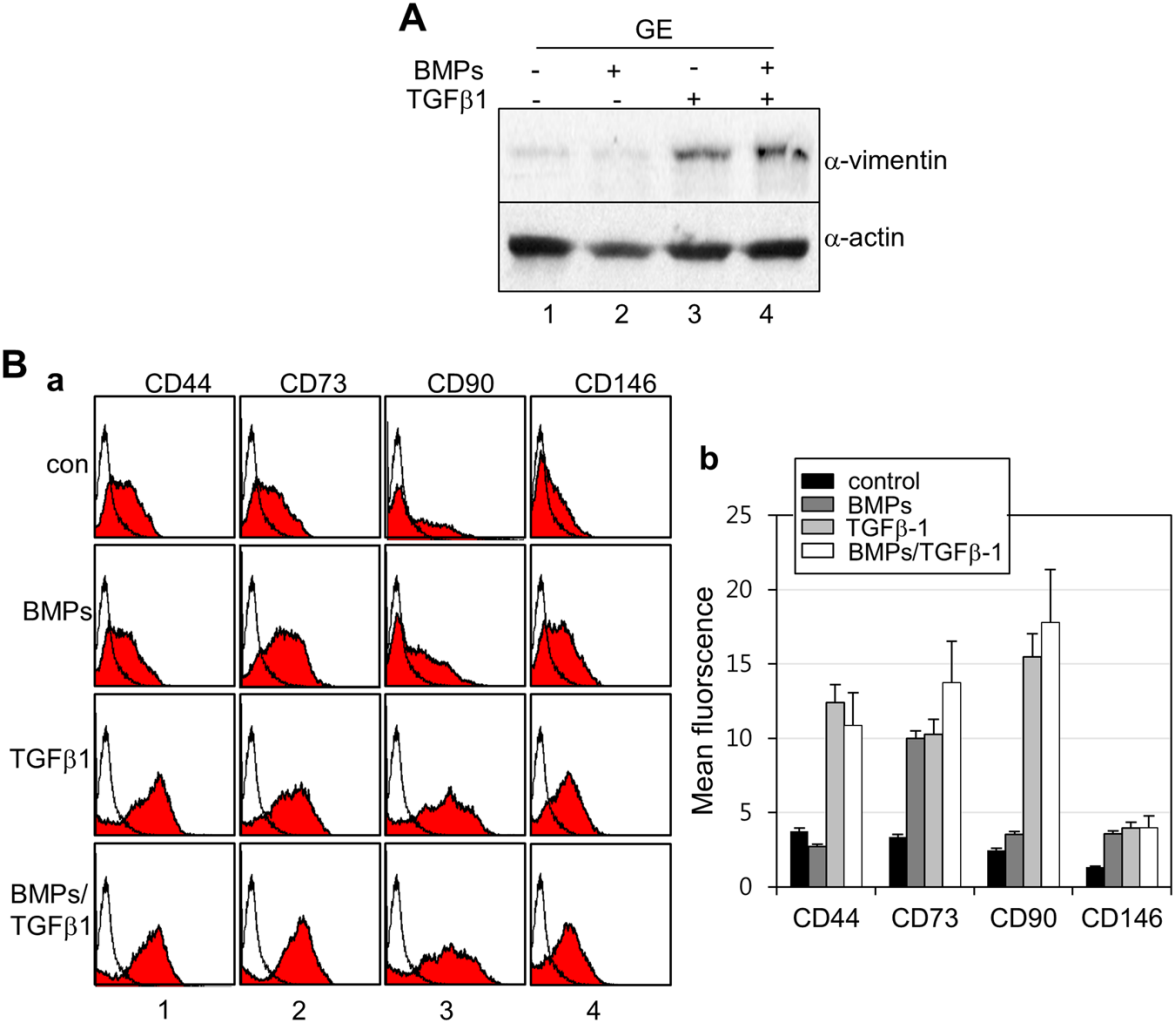
Epithelial-mesenchymal transition is occurred in the gingival epithelial cells during ameloblastic cytodifferentiation. (**A**) Expression of vimentin, a mesenchymal fibroblast marker in cells treated with cytokines for 7 days. Endogenous vimentin (α-vimentin) and actin (α-actin) were detected by western blot analysis. 1, control without treatment; 2, co-treatment with BMP4 & BMP2; 3, treatment with TGFβ-1; 4, co-treatment with BMP4, BMP2, & TGFβ-1. (**B**) Immunophenotyping of the gingival epithelial cells treated with cytokines. Expression of the mesenchymal cell surface was analyzed by FACS analysis after incubation with anti-CD44, anti-CD73, anti-CD90, and anti-CD146 antibodies. *a*, FACS histograms; *b*, mean fluorescence calculated from FACS data. This FACS histogram was representative of one of the three experiments using three individual cultures of gingival epithelial cells.

When gingival epithelial cells were treated with 100 ng/ml BMP4 or 100 ng/ml BMP4 and BMP2 for 7 days, morphological phenotype did not change much from the initial epithelial phenotype (Fig. 3A, panels a–c). However, bipolar fibroblastic phenotypes appeared when gingival epithelial cells were treated with TGFβ-1 alone (Fig. 3A, panel d) and treated with BMP4, BMP2, and TGFβ-1 simultaneously (Fig. 3A, panel e). Under these conditions, the expressions of ameloblast-specific markers such as amelogenin, enamelin, and ameloblastin was notably increased in cells co-treated with BMP4, BMP2, and TGFβ-1 by 1.7, 4.7, and 15.9 times, respectively (Fig. 3B, bar 5 in a–c) compared with those in cells treated individually or with BMPs only (Fig. 3B, bars 2–4 in a–c). Although we also analyzed the mRNA expressions of KLK4 and MMP20, which are known as the enamel specific markers, these gene expressions were much increased by co-treatment with BMP2, 4, and TGFβ-1 (Fig. 3B, g & h). As is the case in ameloblast-specific markers, the expressions of osteogenic markers were also highly increased in cells co-treated with BMP4, BMP2, and TGFβ-1 (Fig. 3B, bar 5 in d–f) than in cells treated individually or with BMPs only (Fig. 3B, bars 2–4 in d–f). Because tooth enamel is a highly mineralized tissue, we analyzed the ALP activity and mineralization efficiency to prove the efficiency of enamel formation in ameloblast-like cells. After treatment with BMPs and/or TGFβ-1 for 7 days, cells were cultured in a medium containing ascorbic acid, β-glycerophosphate, and dexamethasone for the induction of mineralization. Although the ALP activity was not much increased in epithelial cells treated with BMPs or TGFβ-1 only (Fig. 3C, bars 2 & 3), it was highly increased by 2.8 times on co-treatment (Fig. 3C, bars 1 & 4). In addition, when cells were stained by alizarin red for analyzing mineralization efficiency, the intensity was highly increased in cells co-treated with BMPs and TGFβ-1 (Figs 3D, 4 in a & b). These results suggested that gingival epithelial cells are efficiently cytodifferentiated into ameloblast-like progenitors by co-treatment with BMPs and TGFβ-1 in a synergistic manner.

As shown in Fig. 2B, when gingival epithelial cells were treated with TGFβ-1 alone or BMPs and TGFβ-1, the polygonal phenotype was changed into a fibroblastic shape, and the expression of vimentin, a fibroblastic marker, was increased (Fig. 4A, lanes 3 & 4 in upper panel). As expected, the expressions of mesenchymal cell markers, CD44, CD73, CD90, and CD146, were notably increased in cells treated with TGFβ-1 alone or with BMPs and TGFβ-1 (Fig. 4B, a & b). However, the expressions of epithelial stem cell markers, EpCAM, p75NTR, and integrin α-6, were unchanged by treatment with TGFβ-1 alone or with BMPs and TGFβ-1 (data not shown). These findings suggested that gingival epithelial cells are transformed into fibroblastic cells by TGFβ-1 or BMPs and TGFβ-1. Although treatment with TGFβ-1 alone seemed to be sufficient to induce the mesenchymal transition of epithelial cells, the expression of ameloblast-specific genes was efficiently stimulated by co-treatment with BMPs and TGFβ-1 (Fig. 3B). During ameloblastic induction, the activations of Smad and p38 mitogen-activated protein kinase (MAPK) were examined. Both Smad1/5/8 and Smad3 and p38 were highly phosphorylated in cells treated with BMPs alone or BMPs and TGFβ-1 (Supplementary Fig. 1), indicated that the non-canonical MAPK pathway as well as the canonical pathway might be stimulated by BMPs and TGFβ-1 during the epithelial-mesenchymal transition for the ameloblastic cytodifferentiation of epithelial cells.

### Characterization of ameloblast-specific cell surface markers through RNA-Seq based transcriptome analysis

**Figure 5:**
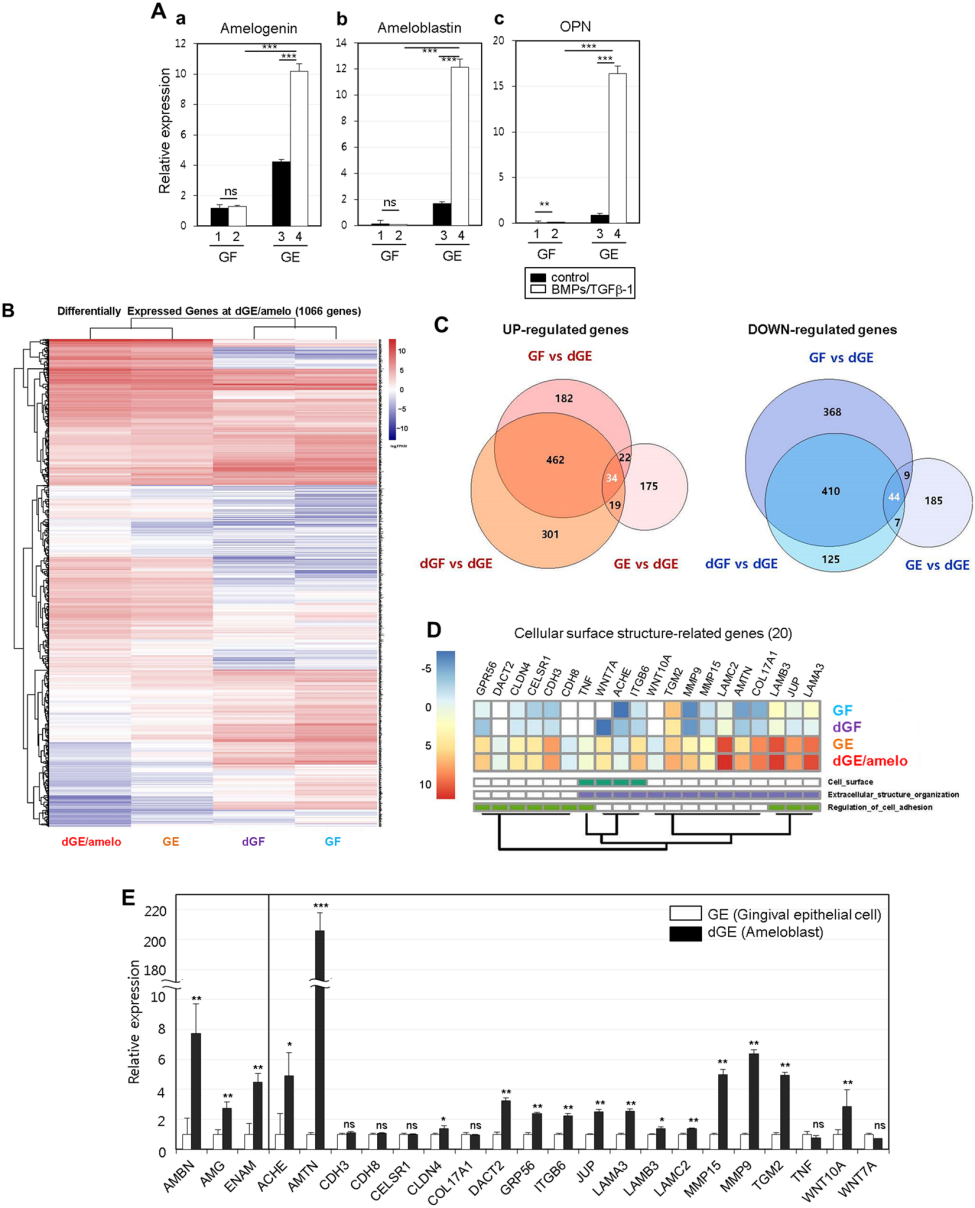
Transcriptome analysis of ameloblast-like cells derived from gingival epithelial cells. (**A**) Validation of molecular differences among the fibroblasts and epithelial cells. Relative mRNA expressions of ameloblastic and osteogenic markers were analyzed by qPCR in different states of gingival cells. For differentiation, cells were treated with BMP4, BMP2, and TGFβ-1 for 7 days. *a*, expression of amelogenin; *b*, expression of ameloblastin; *c*, expression of osteopontin. 1 & 3, gene expression in cells without cytokines; 2 & 4, gene expression in cells treated with cytokines. Statistical significance of *P < 0.1, **P < 0.01, or ***P < 0.001 was determined by using Student t-test from three attempts performed by a limited culture number of 10 individual cultures. (**B**) The number of up- and down-regulated genes identified from the three comparison groups (GF vs dGE, dGF vs dGE, and GE vs dGE). Overlapping areas in Venn diagram represent common genes between the comparison groups. *dGE/amelo*, gingival epithelial cells treated with cytokines; *GE*, gingival epithelial cells without cytokines; *dGF*, gingival fibroblasts treated with cytokines; *GF*, gingival fibroblasts without cytokines. See also Table S2, Figs S1 and S2. (**C**) Heat map of 1007-differentially expressed genes (DEGs) from the pairwise comparison. Rows and columns represent 1007 DEGs (537 up- and 470 down-regulated genes) and profiled samples, respectively. The relative expression was depicted according to the color scale. See also Figs S3, S4, and S5. (**D**) Gene expression profile of the 20 genes selected by association with three major GO categories ‘cell surface’, ‘extracellular structure organization’, and ‘regulation of cell adhesion’. The heat map shows log_2_FPKM values for 20 selected genes (rows) and four different states of cell (columns). Three GO categories were arranged by hierarchical clustering at left columns (cell surface in green, extracellular structure organization in purple, and regulation of cell adhesion in olive). (**E**) Confirmation of individual gene expression by qRT-PCR. Statistical significance of *P < 0.1, **P < 0.01, or ***P < 0.001 was determined by using Student t-test. *GE*, gingival epithelial cells without cytokines; *dGE/amelo*, gingival epithelial cells treated with cytokines.

To perform a comparative analysis of the gene expression profiles during ameloblastic differentiation, four different states of cells were used: gingival fibroblasts (GF), gingival fibroblasts treated with BMPs/TGFβ-1 (dGF), gingival epithelial cells (GE), and gingival epithelial cells treated with BMPs/TGFβ-1 (dGE/amelo). For verification of the cell states before analysis, ameloblast-specific markers were validated by qPCR. As expected, the expressions of amelogenin, ameloblastin, and OPN in GF were lower than those in GE without treatment with cytokines (Fig. 5A, bars 1 & 3 in a–c). In contrast, treatment with BMPs/TGFβ-1 did not induce gene expression in GF (Fig. 5A, bars 1 & 2 in a–c), while the expressions of amelogenin, ameloblastin, and OPN were increased in GE treated with BMPs/TGFβ-1 (Fig. 5A, bars 3 & 4 in a–c). On the basis of the above results, GE treated with BMPs/TGFβ-1 was considered as ameloblast-like cells. RNA-Seq was performed to compare the gene expression profiling of the four different states of cells. According to Ensembl gene annotation, the expression of 18,313 genes in the four different cell types were identified, and the genes with log2FPKM ≥ 1 which are mainly expressed in four different cell states were used for hierarchical clustering analysis (Supplementary Table S2 and Supplementary Fig. S1A). The levels of gene expression showed a uniform distribution in all cell types, indicating the reliability of our data (Supplementary Fig. S1B). We established three pairwise comparisons as follows: dGE/amelo vs. GF, dGE/amelo vs. dGF, and dGE/amelo vs. GE. Totals of 1531 genes (700 up- and 831 down-regulated) and 1402 genes (816 up- and 586 down-regulated) were differentially expressed in dGE/amelo in comparison with GF and dGF, respectively (Fig. 5B and Supplementary Fig. S2A,B). In the dGE/amelo vs. GE comparison, we observed 495 DEGs (250 up- and 245 down-regulated) (Fig. 5B and Supplementary Fig. S2C). Five hundred thirty-seven of 1195 genes and 470 of 1148 genes showed higher and lower expression, respectively, in dGE/amelo rather than in the other cells (Fig. 5B). Based on the gene expression data, we performed hierarchical clustering analysis using a total of 1007 DEGs in dGE/amelo (Fig. 5C). Particularly, 34 up- and 44 down-regulated genes were shared by all comparison groups (Fig. 5B and Supplementary Fig. S3). The GO classification for 470 down-regulated genes is related to the processes of “cell cycle”, “DNA replication”, “nuclear division”, and “spindle organization” (Supplementary Fig. S4), suggesting that gingival epithelial cells could not undergo proliferation when cells were stimulated with cytokines. The 238 of 537 up-regulated genes were significantly enriched and reclassified into eight major GO categories, namely, “cell surface”, “stimulus response”, “cytokine production”, “developmental process”, “extracellular organization”, “signal transduction”, “cell adhesion”, and “cell motility” (Supplementary Fig. S5). In contrast to the downregulation of proliferation-related genes, genes associated with the developmental process were up-regulated in dGE/amelo. We focused on GO categories such as “cell surface”, “extracellular organization”, and “cell adhesion” for the identification of ameloblast-specific cell surface markers. By analyzing the transcriptome signatures and biological implications of DEGs, we were able to identify 20 genes known as cell surface molecules, which might be associated with amelogenesis: *ACHE*, *AMTN*, *CDH3*, *CDH8*, *CELSR1*, *CLDN4*, *COL17A1*, *DACT2*, *GPR56*, *ITGB6*, *JUP*, *LAMA3*, *LAMB3*, *LAMC2*, *MMP9*, *MMP15*, *TGM2*, *TNF*, *WNT7A*, and *WNT10A* (Fig. 5D), and their expressions were checked again through qRT-PCR (Fig. 5E). As positive markers of ameloblasts, the expressions of ameloblastin, amelogenin, and enamelin were higher in ameloblast-like cells than in gingival epithelial cells. Most of the 20 markers were increased in ameloblast-like cells, although the expression changes of *COL17A1*, *TNF*, and *WNT7A* were not statistically significant.

## Discussion

So far, it has been difficult to study amelogenic differentiation because ameloblasts disappear due to apoptosis during tooth eruption and enamel epithelium remains fully mineralized in the human tooth. For this reason, most studies on amelogenic differentiation have been performed using rat and mouse incisors. Although the epithelial cells having the capacity to differentiate into ameloblasts have been cultured from root epithelium cell lineages such as HERS/ERM^26,27^, these tissues are generally not easy to obtain from intact teeth extracted from human adults. To secure human ameloblasts, at first, we developed the epithelial cell culture procedure using human dental gingival tissue, which is a relatively accessible dental tissue. Gingival fibroblasts and gingival epithelial cells turned out to have completely different cellular and biochemical features (Figs 2 and 5A). Interestingly, although these gingival epithelial cells were still expressing mesenchymal markers (Fig. 2C, c & d), this phenomenon was also seen in cells originating from ERM from periodontal ligament^21^. For ameloblastic cytodifferentiation, gingival epithelial cells were treated with BMP2, BMP4, and/or TGFβ-1. Treatment with TGFβ-1 alone induced the epithelial-mesenchymal transition (Fig. 3A & bar 4 in B), but when co-treated with BMPs and TGFβ-1, the expression of ameloblast-specific genes was apparently increased, as was the ALP activity (Fig. 3B,C).

Although ameloblast-like phenotypes were induced in gingival epithelial cells, we were unable to conclude that gingival epithelial cells treated BMPs/TGF could have the same molecular signature as real human ameloblasts. It is impossible for culture the real ameloblasts from adult tooth sample, but there may be another way to isolate ameloblasts from tooth germ of third molar.

A recent study demonstrated that epithelial cells undergo the epithelial-mesenchymal transition in the presence of TGFβ-1 during differentiation to hard tissues such as cementum^21,27,28^. Our data indicated that BMPs accelerate the epithelial-mesenchymal transition induced by TGFβ-1 and ameloblastic cytodifferentiation. TGFβ-1 and BMPs have a synergistic effect during amelogenesis (Figs 3 and 5A).

Previously, it was reported that TGFβ-1 suppresses the process of hard tissue formation stimulated by BMPs in mesenchymal progenitor cells^29,30^, suggesting that the BMP response might differ between mesenchymal and epithelial cells. The Activation of both Smad1/5/8 and Smad2/3 pathways is found in diverse epithelial lineages^31^. Smad pathways were stimulated when cells were treated with TGFβ-1/BMPs, but the phosphorylation of each Smad was not synergistically increased by co-treatment. In addition to the canonical Smad pathway, p38 phosphorylation was increased in epithelial cells treated with TGFβ-1/BMPs (Supplementary Fig. 1), suggesting that the MAPK pathway is also involved in oral epithelial and enamel formation.

Exploration of progenitor-specific cell surface markers and cell purification using these markers is important to supply a specific stem cell source for tissue regeneration. Although decoy immunization has many advantages in terms of directly obtaining the surface antibody^32^, this method has a downside that a large number of cells are needed. Because of this limitation, we performed RNA-Seq to analyze the gene expression profile of ameloblast-like cells. To further identify which genes are closely related to the character of the cell surface structure on ameloblasts, we focused on 20 genes associated with the cell surface, extracellular structure, and cell adhesion (Fig. 5D). ACHE, CELSR1, CLDN4, DACT2, LAMA3, LAMC2, WNT7A, and WNT10A have been reported in previous studies to have direct and indirect relevance to amelogenesis, enamel formation, epithelial cell adhesion, or stabilization of the extracellular matrix in tooth epithelium^33–46^. MMP9 and MMP15 are possibly involved in controlling enamel formation^47,48^, although information such as phenotype of MMP9 or MMP15 deficiency in animal model or mutation in humans was not reported so far. TNF is known to promote the transcription of the human amelotin (AMTN) gene encoding an enamel protein^49–51^, indicating that TNF expression is correlated with increased AMTN gene expression and leads to amelogenesis in gingival epithelial cells. Previously, an effort was made to characterize the transcriptome profile of ameloblasts, which were located in tooth buds of human fetuses and captured by laser-dissection^52^. This study reported that 21 genes were significantly increased in pre-secretory ameloblasts than in odontoblasts and secretory ameloblasts and 4 genes were specifically expressed in secretory ameloblasts based on RNA microarray data. Some of them were given a baseline of genes expressed by ameloblasts: dentin matrix protein-1, ameloblastin (expressed in pre-secretory ameloblasts), enamelin, and amelogenin (expressed in secretory ameloblasts), suggesting that the ameloblast-like cells that are differentiated from gingival epithelial cells in this study have the characteristics of ameloblasts (Fig. 5A,E). Although further validation is required, these 20 gene products and their specific antibodies could be useful as ameloblast-specific cell surface markers in future studies. The 20 genes selected are indeed important in amelogenesis and may be involved in amelogenesis imperfecta.

## Materials and Methods

### Cell cultures

Intact third molar teeth were collected from ten dental surgery patients aged 19~29 years old under guidelines approved by the Dankook Dental Hospital, and the informed consent for all experiments using extracted teeth was obtained from all participants. Gingival tissues were separated from gum part on extracted teeth and were treated with 3 mg/ml of collagenase (Sigma-Aldrich) and 4 mg/ml of dispase (Sigma-Aldrich). The primary gingival cells were grown outward from the sliced tissues in α-MEM containing 20% FBS (Hyclone). The gingival fibroblasts (GF) were dominantly grown out by continuous culture in the same media^23^. For isolation of gingival epithelial cells (GE), cells grown outward from tissues were trypsinized and washed with Keratinocyte Basal Medium (KBM, Lonza), and were transferred into Keratinocyte Growth Medium (KGM, Lonza). To collect only GE, the selective trypsinization was performed with modification in previous procedure^53^. GF were thrown out by treatment with low concentration of trypsin (0.062% trypsin/0.552 mM EDTA), and remaining GE were harvested by trypsinization using 0.25% trypsin/2.21 mM EDTA. Summary of cell culture procedure was shown in Fig. 1. Gingival epithelial cells collected were verified by immunophenotyping using epithelial and mesenchymal cell markers as mentioned below. The data in this report were originated from a cell line or from the average value of individual cell lines (see Figure legends). For ameloblastic cytodifferentiation, cells were treated with 100 ng/ml of BMP4 and BMP2, and/or 10 ng/ml of TGFβ-1 for 7 days in KGM.

### Flow cytometric analysis

Cells were resuspended in PBA (PBS containing 0.5% BSA) and incubated with FITC- or PE-conjugated antibodies for 1 hour on ice. The antibodies used are as follows: FITC-integrin α-6, FITC-EpCAM, FITC-p75NTR, PE-CD44, PE-CD73, PE-CD90, and PE-CD146 antibodies purchased from BD Bioscience. The fluorescent signals were measured by using FACSCalibur flow cytometer (BD Bioscience), and were analyzed using WinMDI software.

### Western blot analysis

Cell lysate was prepared by using NP-40 lysis buffer (20 mM Tris-HCl, pH8.0, 150 mM NaCl, 0.5% NP-40, 2 mM EGTA, 1 mM EDTA, 1 mM Na_3_VO_4_, 10 mM NaF, 20 mM *p*-nitrophenol phosphate, and protease inhibitors). Total proteins were separated by SDS-PAGE and were transferred to PVDF membrane. The membrane was blocked with 5% skim milk or BSA in TBST (0.1% Tween 20, 150 mM NaCl, 10 mM Tris-HCl, pH7.6). After blocking, membranes were incubated with the primary antibody and the HRP-conjugated secondary antibody. Signals were visualized using the ECL^TM^ system (Amersham Biosciences). Anti-phospho-Smad1/5/8, -Smad1, -phospho-Smad3, and -Smad3 antibodies were purchased from Cell Signaling Technology. Anti-phospho-p38, -p38, and -vimentin antibodies were purchased from Santa Cruz Biotechnology.

### Quantitative Real Time-PCR analysis

Total RNA was purified from cells using Easy-Spin^TM^ Kit (iNtRON), and used for cDNA synthesis using ReverTra Mix^TM^ (Toyobo). The gene expression levels were analyzed by quantitative real time-PCR (qRT-PCR) using StepOn^TM^ system (Applied Biosystems) with SYBR Green Supermix^TM^ (Bio-Rad). Primers used were indicated at Supplementary Table S1. GAPDH was used as a control to normalize the variability in target gene expression. During qRT-PCR, a dissociation curve was constructed in the range of 65 °C to 95 °C, and the cycling parameters were followed as: 1 cycle for 1 min at 95 °C, 40 cycles for 15 sec at 95 °C, and 1 cycle for 1 min at 60 °C. The threshold cycle was obtained and the relative comparison of each target gene was analyzed.

### Mineralization and alkaline phosphatase (ALP) assay

Cells were treated with the BMPs and TGF for 7 days, followed by treatment with osteogenic reagents (50 μg/ml ascorbic acid, 10 mM β-glycerophosphate, and 5 μM dexamethasone) for another 7 days. After 7 days of osteogenic induction, ALP activity was analyzed using ALP assay kit (BioVision). Briefly, cells were collected and disrupted by sonication, and after centrifugation, the supernatant incubated with *p*NPP substrate at 25 °C in dark. Absorbance was measured at 405 nm. For mineralization analysis, alizarin red staining was performed after the osteogenic induction. Cells were fixed with 4% paraformaldehyde for 15 min, washed with PBS, and treated with 2% alizarin red S (pH 4.5, Sigma). For quantification, plate was incubated with 10% acetic acid for 30 min at RT and heated for 10 min at 85 °C. Then, supernatants were neutralized by 10% ammonium hydroxide.

### Library construction and analyses of differentially expressed genes (DEGs) in RNA-Seq analysis

After purification of total RNA, RNA purity was estimated by using Agilent 2100 Bioanalyzer (Agilent Technologies), and mRNA was enriched by oligo-dT magnetic bead. After cDNA synthesis, the samples were sequentially subjected to end-repair and addition with poly-A and adaptors using TruSeq^TM^ RNA prep Kit (Illumina). cDNA fragments of 400–500 bps were separated on BluePippin^TM^ system (Sage Science) and constructed into final library. Sequencing was performed in the paired-end sequencing mode using an Illumina Hiseq2500 sequencer (Illumina). Prior to read mapping, the raw reads with low-quality were filtered through in-house scripts as previous reported^54^. The qualified reads were implicated in alignment to the human genome (Ensembl release 72) using TopHat version 2.1.0^55,56^, and uniquely mapped read pairs were used for the further analysis. The levels of gene expression with fragments per kilobase of exon per million fragments (FPKM) were calculated, and DEGs were determined using CUFFLINKS v.2.2.1^57^. For each of the three pairwise comparisons, genes displaying log_2_FC(fold-change) ≥1 and p-value -v0.05 were handled. Gene ontology (GO) analysis of DEGs was performed using Metascape (http://metascape.org/gp/index.html). Further, clustering analysis of DEGs was performed based on the log_2_FPKM values and the heat map was generated using Pheatmap v1.0.8 with the popular clustering and hierarchical clustering method functions.

## Supplementary information

Supplemental infornation
