# Supplementary material for: Amelogenic transcriptome profiling in ameloblast-like cells derived from adult gingival epithelial cells
Source: Sci Rep. 2019 Mar 6;9:3736. doi: 10.1038/s41598-019-40091-x (PMC6403286; doi:10.1038/s41598-019-40091-x)
Supplement: Supplementary file 1 — Supplemental infornation [file 41598_2019_40091_MOESM1_ESM.pdf]

**Supplementary Information**

# Amelogenic transcriptome profiling in ameloblast-like cells derived from adult gingival epithelial cells

Sun-Yi Hyun[1], Seyoung Mun[1,2], Kyung-Jung Kang[1], Jong-Chan Lim[1], Shin-Young Kim[1], Kyudong Han[1,2], and Young-Joo Jang[1,*]

**Supplementary Table 1. Primers used for quantitative real time PCR**

| Target gene | Primer sequences |
|---|---|
| Amelogenin | Forward-5’-TGCCTCTACCACCTCATC-3’<br>Reverse-5’-GATGTGGTGATGAGGCT-3’ |
| Ameloblastin | Forward-5’-AAAACCCAGCTTTCCTTACA-3’<br>Reverse-5’-AACATCAGCTAATTCAGGGG-3’ |
| Enamelin | Forward-5’-CCACCACCTATAGAGAGCAG-3’<br>Reverse-5’-AATAGGGCTGGTTACCTTTC-3’ |
| Bone sialo protein (BSP) | Forward-5’-TACCGAGCCTATGAAGATGA-3’<br>Reverse-5’-CTTCCTGAGTTGAACTTCGA-3’ |
| Osteocalcin(OCN) | Forward-5’-TGAGTCCTGAGCAGCAG-3’<br>Reverse-5’-TCTCTTCACTACCTCGCT-3’ |
| Osteopontin(OPN) | Forward-5’-GTGGGAAGGACAGTTATGAA-3’<br>Reverse-5’-CTGACTTTGGAAAGTTCCTG-3’ |
| GAPDH | Forward-5’-GTATGACAACAGCCTCAAGAT-3’<br>Reverse-5’-CCTTCCACGATACCAAAGTT-3’ |

**Supplementary Table 2. Summary statistics for RNA sequencing data.** An average of approximately 6.2 million raw reads was produced and at least 5.92 Gb of clean data, accounting for more than 95.73 % of the raw data, was obtained in each cell state. After the clean reads had been mapped to the reference sequence of Genome Reference Consortium GRCh37 (hg19), we reached an average of more than 92% read pairs being uniquely mapped on the human genome.

| # | Case | Sample Name | Raw data | After filtering (%) | Mapped reads (%) | Uniquely mapped reads (%) | Ensembl 72 (23,362 coding genes) | |
|---|---|---|---|---|---|---|---|---|
| | | | | | | | Expressed genes (FPKM > 0) | Unexpres sed genes |
| 1 | Untreated gingival fibroblast cell | GF | 70,295,202 | 67,418,406 (95.9%) | 63,697,140 (94.5%) | 62,568,932 (92.8%) | 16,343 | 7,019 |
| 2 | Untreated gingival epithelial cell | GE | 60,652,926 | 58,351,214 (96.2%) | 55,036,859 (94.3%) | 53,678,055 (92.0%) | 16,770 | 6,592 |
| 3 | Differentiated gingival fibroblast cell | dGF | 62,745,646 | 59,641,910 (95.1%) | 56,447,189 (94.6%) | 55,416,372 (92.9%) | 15,691 | 7,671 |
| 4 | Differentiated gingival epithelial cell (Ameloblast) | dGE /amelo | 65,911,514 | 63,053,634 (95.7%) | 59,441,469 (94.3%) | 58,090,392 (92.1%) | 16,781 | 6,581 |

**Supplementary Figure S1**

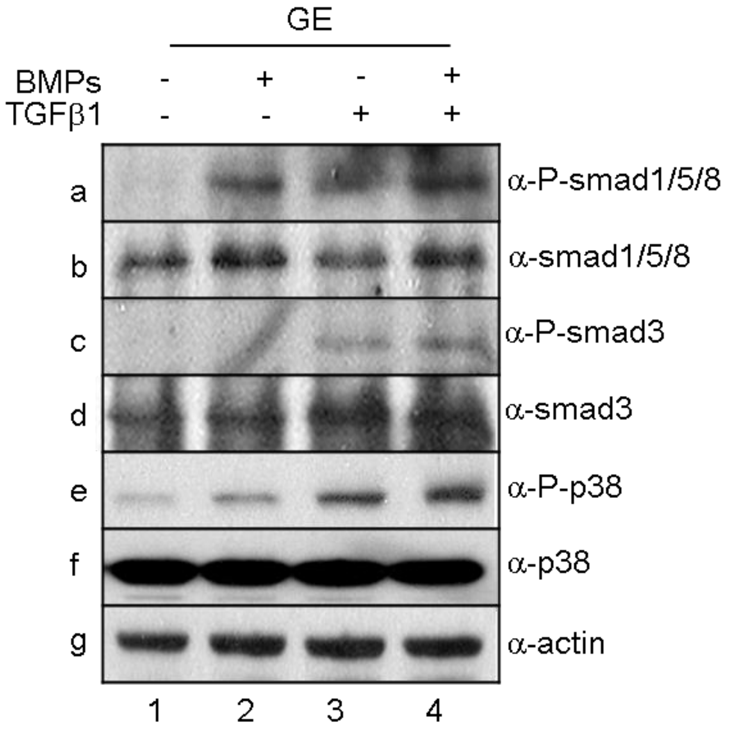


**Supplementary Figure S1. Smads and p38 MAPK signaling pathways are involved in ameloblastic cytodifferentiation of the gingival epithelial cells.** Activation of the signal pathways was indicated as the phosphorylation of specific downstream target proteins. The phosphorylation of Smad1/5/8, Smad3, or p38 was detected by western blot analysis with the phosphorylation-specific antibody (*a*, *c* & *e*). Total Smad1/5/8, Smad3, or p38 was detected by western blot analysis with anti-smad1/5/8, anti-smad3, or anti-p38 antibody, respectively (*b*, *d* & *f*). 1, control without treatment; 2, co-treatment with BMP4 & BMP2; 3, treatment with TGFβ-1; 4, co-treatment with BMP4, BMP2, & TGFβ-1.

**Supplementary Figure S2**

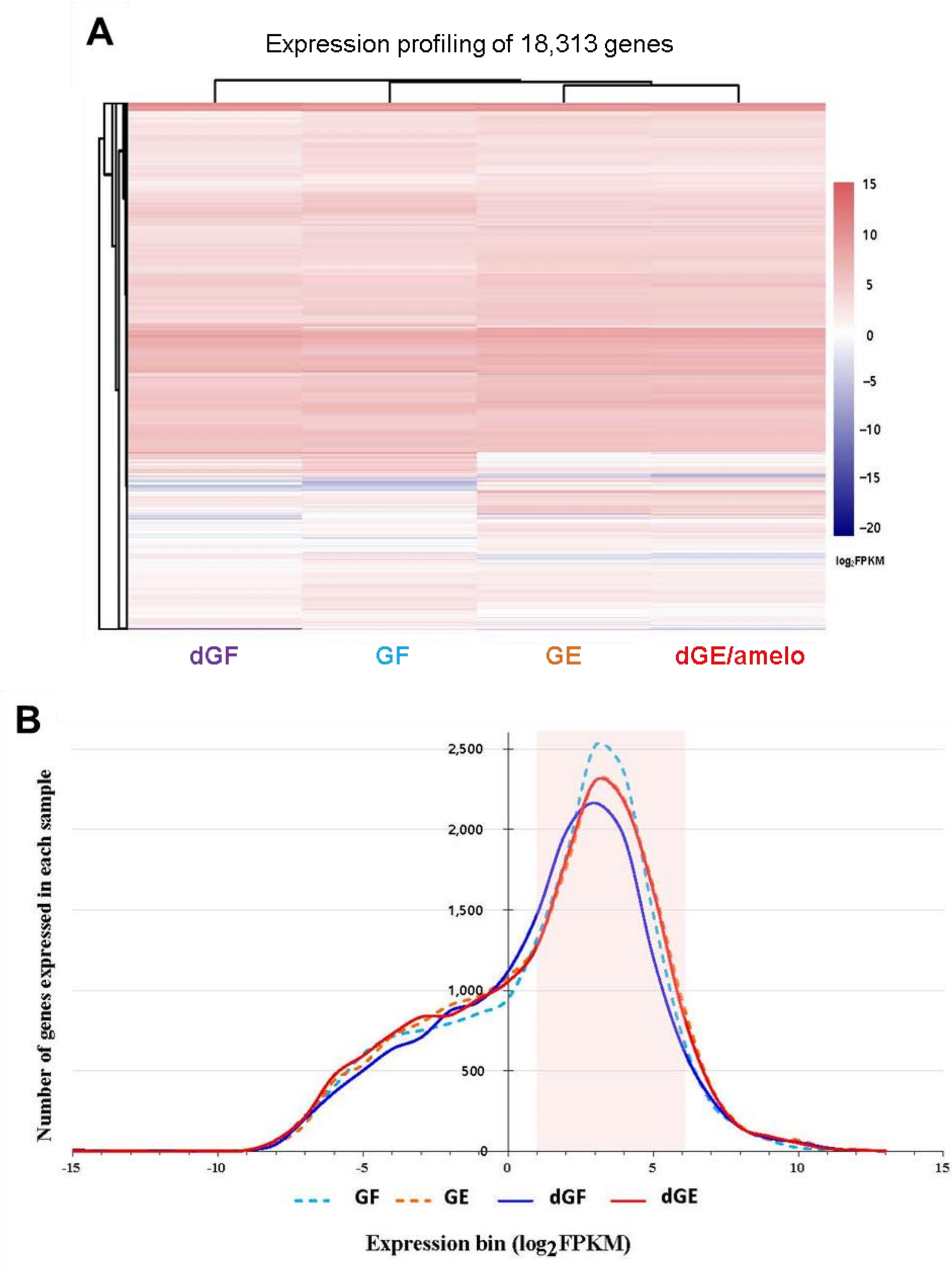


**Supplementary Figure S2. Bioinformatic analysis for gene expression profile. (A)** Heat map of global expression of 18,313 genes in all cells. To demonstrate the source of variance in samples, the correlation between dGE/amelo and the rest was measured, based on

Pearson's correlation coefficient between the $log_2$FPKM values of genes. The transcriptome data for GE showed a high degree of correlation (97.8 %) with dGE, whereas the Pearson's correlation coefficients of GF and dGF were 80.3 % and 83.1 %, respectively. The relative expression was depicted according to the color scale on right. Red and blue indicates up-regulation and down-regulation, respectively. **(B)** Distribution of FPKM values for the identified genes expressed in each cell. The X- and Y-axis indicated the number of expressed transcripts and log-transformed FPKM values of transcripts (FPKM > 0) from samples, respectively. The pink box denotes the majority of transcripts ($1 < log_2$FPKM $< 6$).

**Supplementary Figure S3**

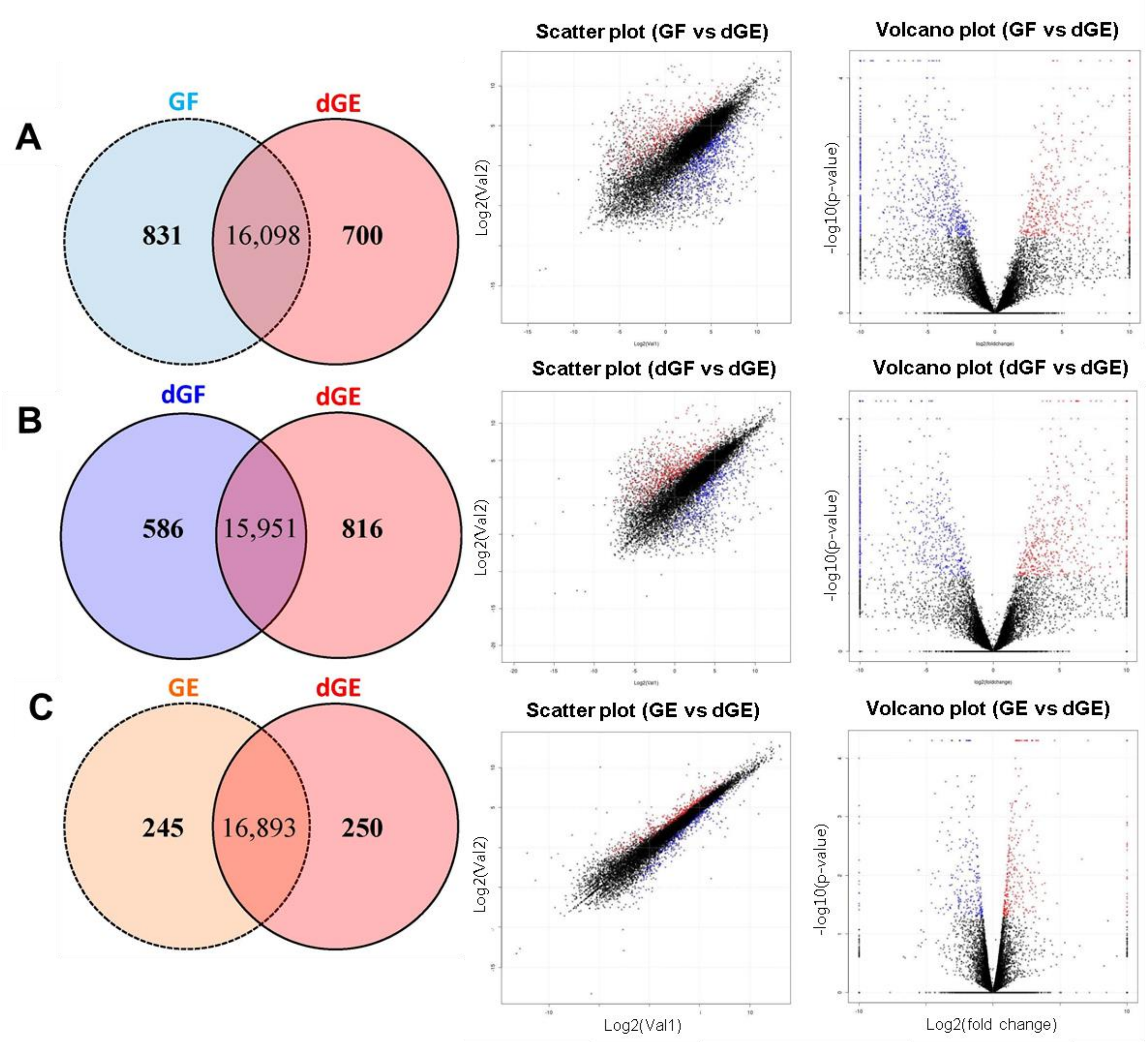


**Supplementary Figure S3. Identification and statistical comparison of differentially expressed genes.** Venn diagrams for each comparison between GF and dGE **(A)**, dGF and dGE **(B)**, and GE and dGE **(C)** showed the distribution of genes corresponding to log2FC ≥ 2 and statistically significant (t-test *p*-value < 0.05). The FPKM scatter plots and the Volcano plots constructed according to pairwise comparison between samples. Red and blue dots in the scatter plots and the Volcano plots represent the significantly up- and down-regulated genes in dGE (*p*–value < 0.05 and log2FC ≥ 2). The middle line in black dots indicated no difference at the mean expression values between three comparison groups.

**Supplementary Figure S4**

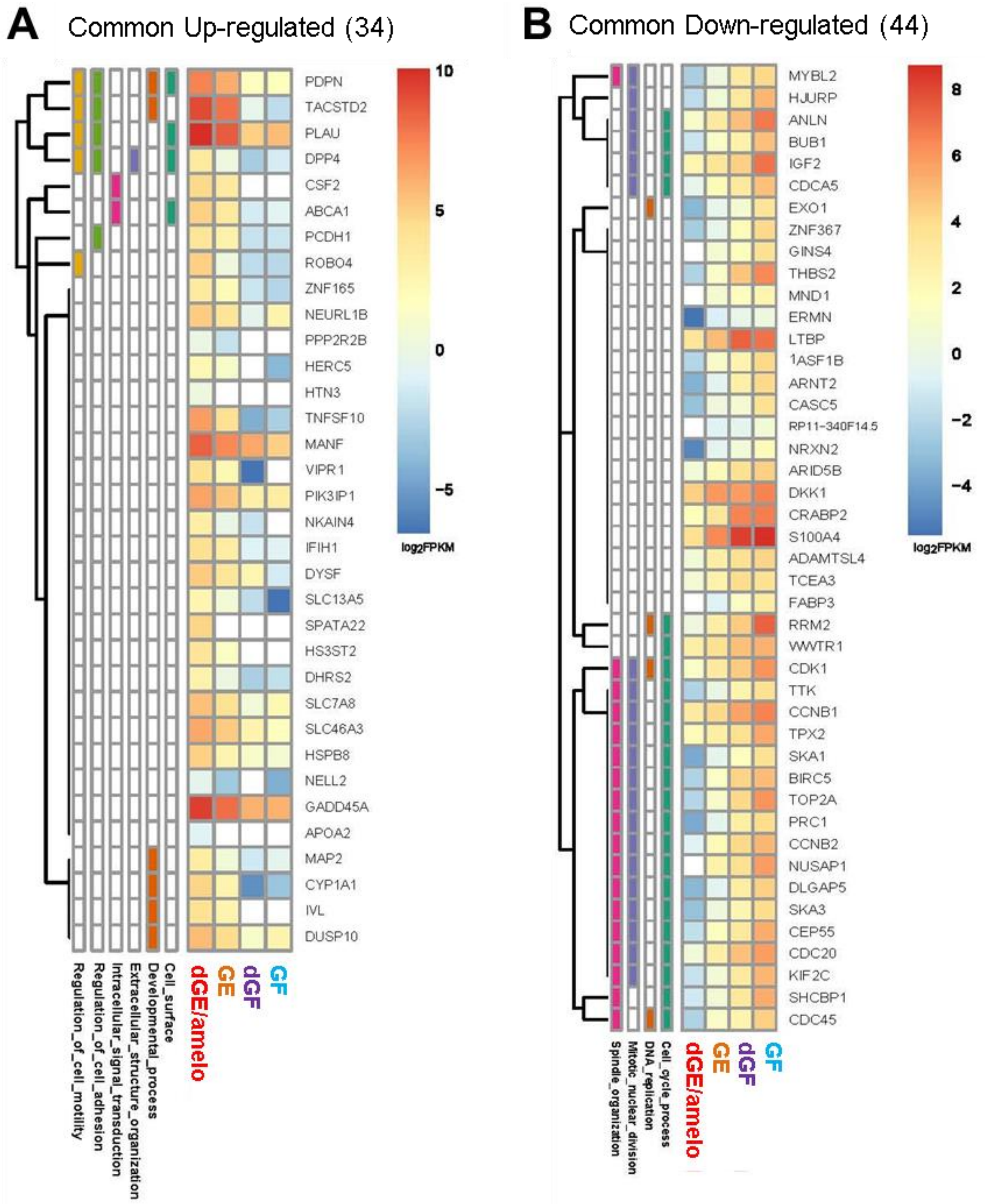


**Supplementary Figure S4. Heat map of the expression profiles of the common 34 up- and 44 down-DEGs from the comparison groups.** The heat map showed the expression level of the common 34 up-regulated **(A)** and 44 down-regulated **(B)** genes based on $log_2$FPKM values (rows). Major enriched GO categories, in which these common DEGs are involved, were arranged by hierarchical clustering at left columns.

**Supplementary Figure S5**

A

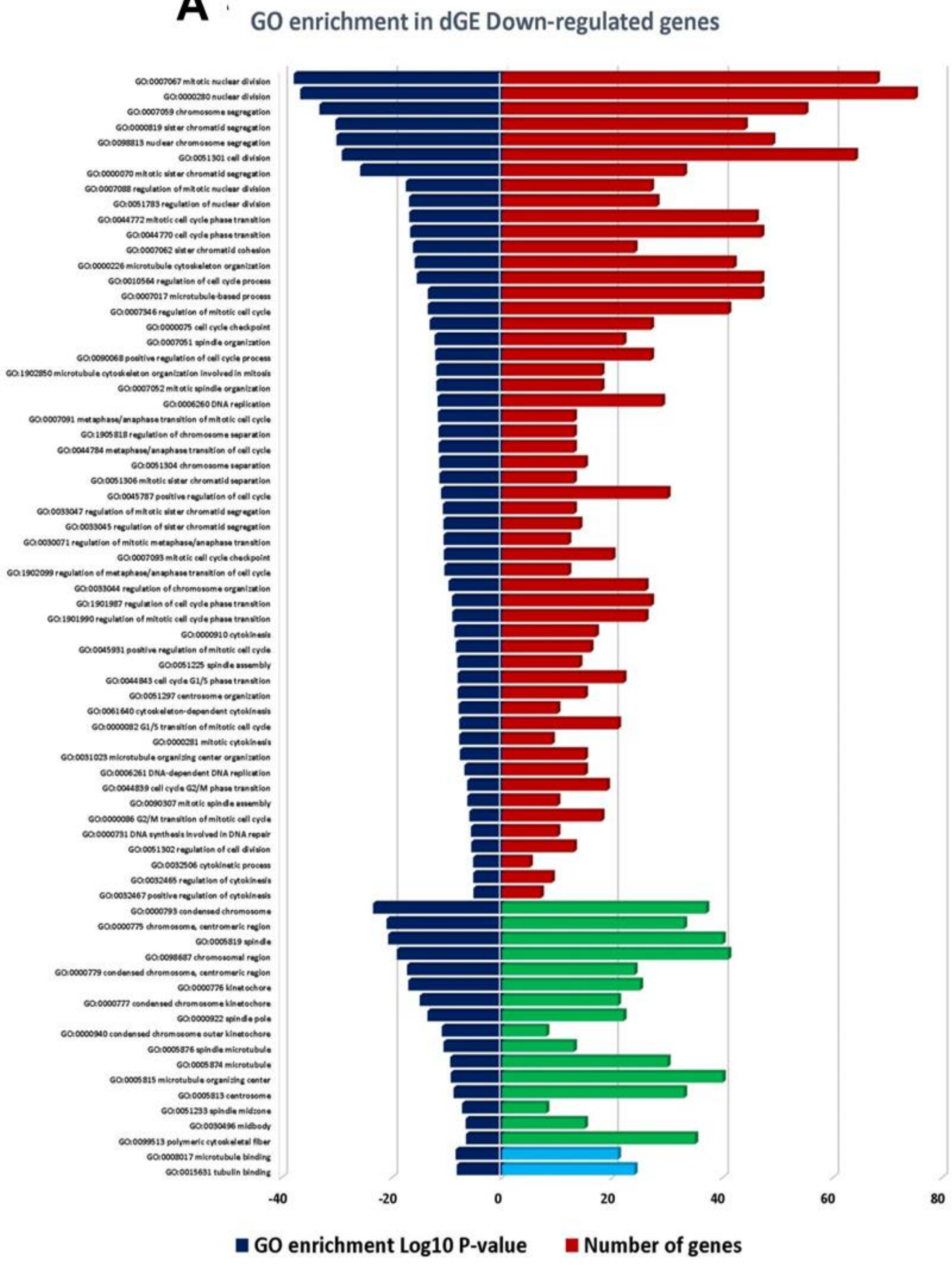

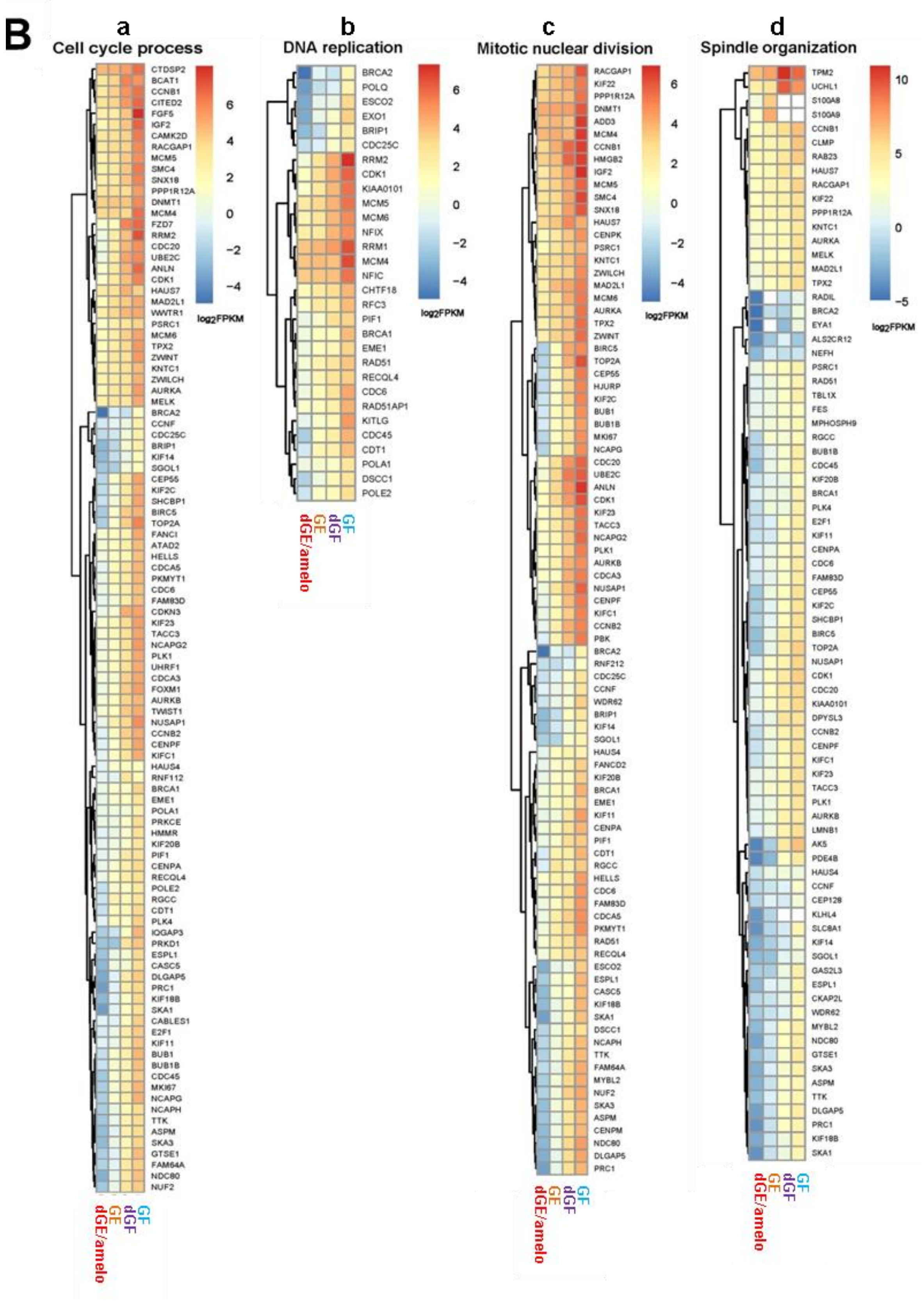


**Supplementary Figure S5. Gene Ontology enrichment analysis and the expression profiles of down-regulated genes in dGE/ameloblast induced by cytokines. (A)** Gene

functional classification in 470 down-regulated genes was performed using the metascape tool. The X-axis in left and right side represented the significance level (scored as $-\log_{10}(p$-value)) and number of genes that map to the corresponding GO terms, respectively. The Y-axis denoted the related functional GO terms (biological process in red, cellular component in green, and molecular function in sky blue). Of the 470 down-regulated genes, 148 were significantly associated with 70 GO terms (52 biological processes, 16 cellular components, and 2 molecular functions). **(B)** Heat map of down-regulated genes belonged to four major GO categories: ***a,*** Cell cycle process; ***b,*** DNA replication; ***c,*** Mitotic nuclear division; ***d,*** Spindle organization.

**Supplementary Figure S5**

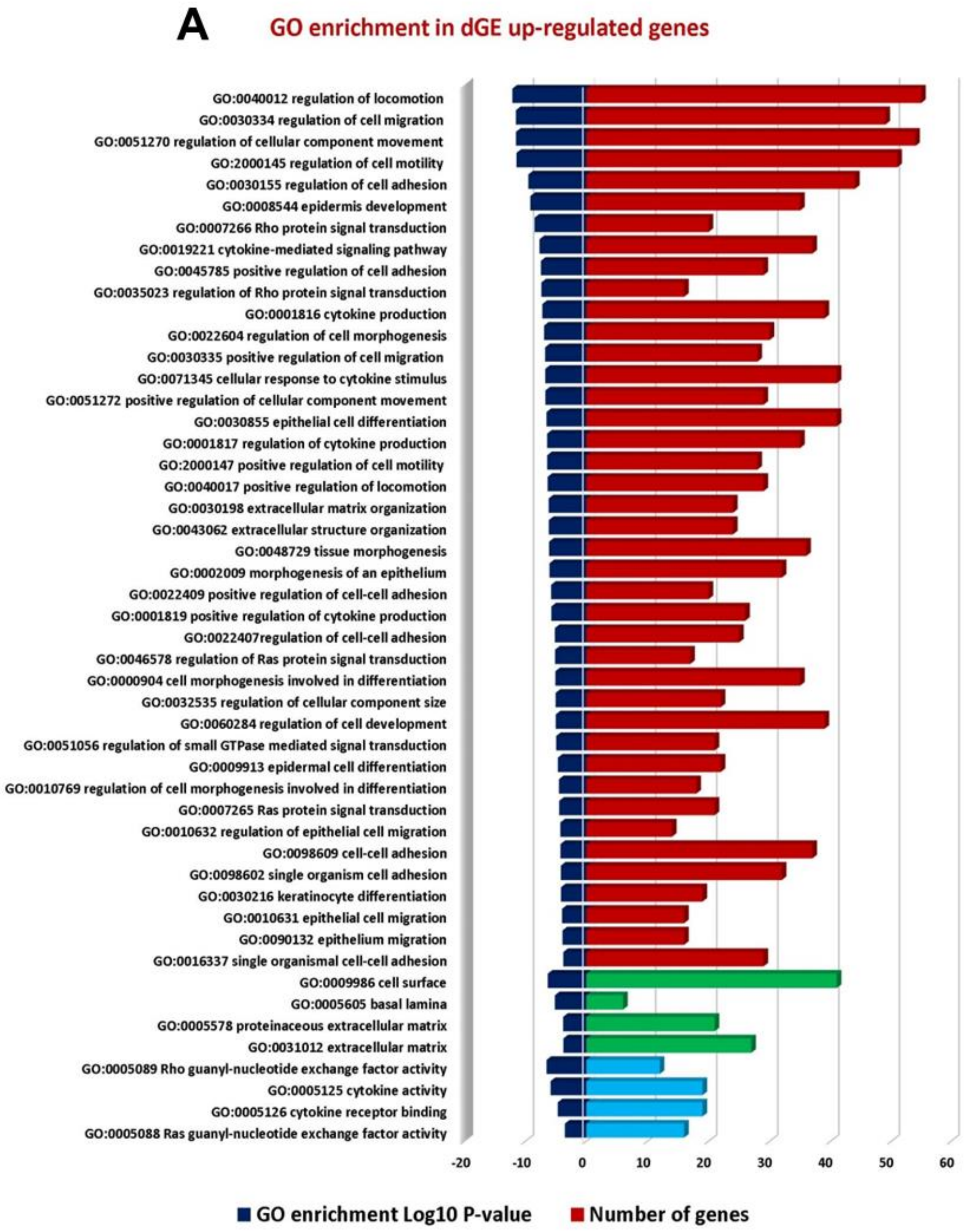

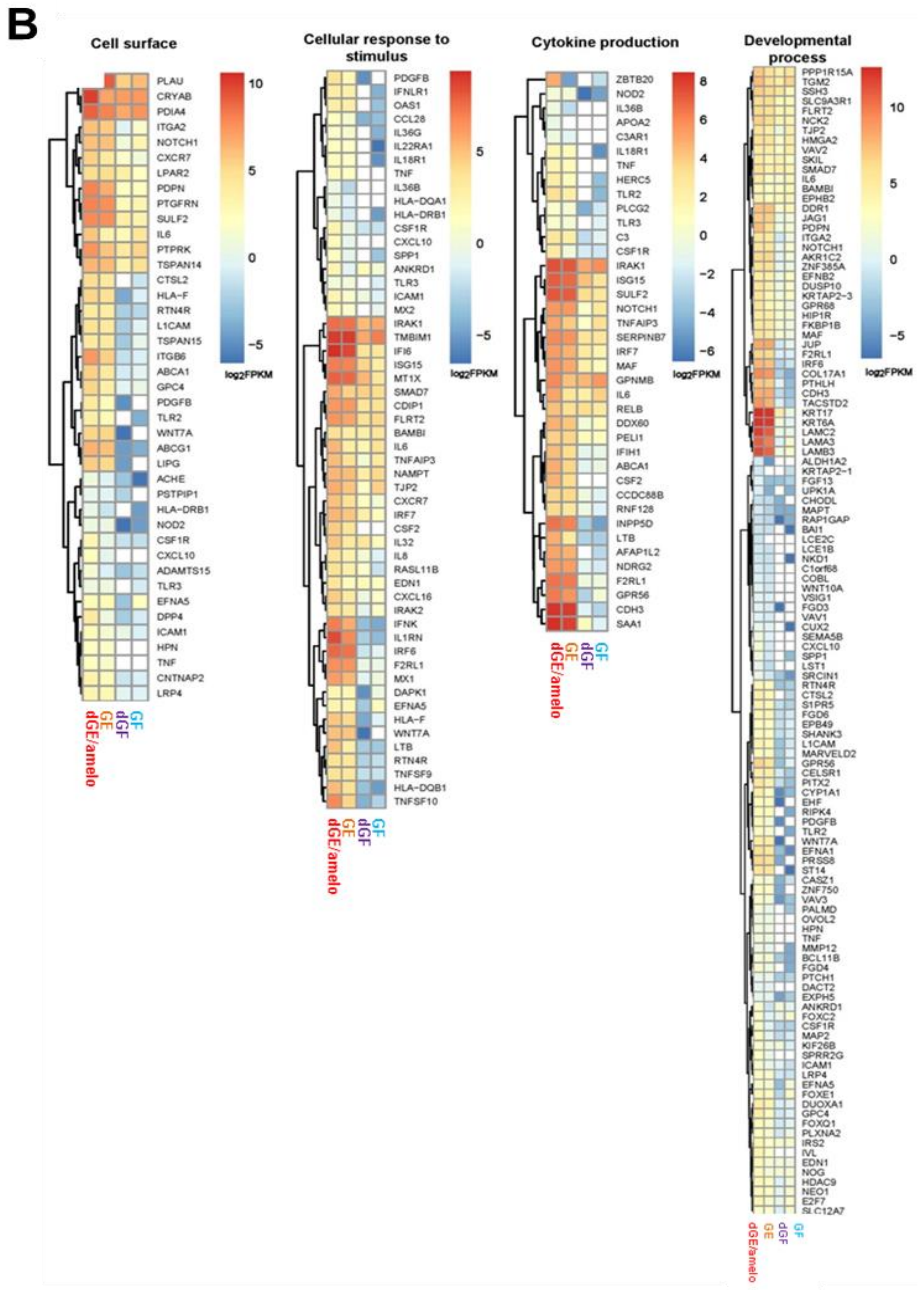
B
Cell surface
PLAU
CRYAB
PDIA4
ITGA2
NOTCH1
CXCR7
LPAR2
PDPN
PTGFRN
SULF2
IL6
PTPRK
TSPAN14
CTSL2
HLA-F
RTN4R
L1CAM
TSPAN15
ITGB6
ABCA1
GPC4
PDGFB
TLR2
WNT7A
ABCG1
LIPG
ACHE
PSTPIP1
HLA-DRB1
NOD2
CSF1R
CXCL10
ADAMTS15
TLR3
EFNA5
DPP4
ICAM1
HPN
TNF
CNTNAP2
LRP4
10
5
0
-5
log2FPKM
dGE/amelo
GE
dGF
GF
Cellular response to stimulus
PDGFB
IFNLR1
OAS1
CCL28
IL36G
IL22RA1
IL18R1
TNF
IL36B
HLA-DQA1
HLA-DRB1
CSF1R
CXCL10
SPP1
ANKRD1
TLR3
ICAM1
MX2
IRAK1
TMBIM1
IFI6
ISG15
MT1X
SMAD7
CDIP1
FLRT2
BAMBI
IL6
TNFAIP3
NAMPT
TJP2
CXCR7
IRF7
CSF2
IL32
IL8
RASL11B
EDN1
CXCL16
IRAK2
IFNK
IL1RN
IRF6
F2RL1
MX1
DAPK1
EFNA5
HLA-F
WNT7A
LTB
RTN4R
TNFSF9
HLA-DQB1
TNFSF10
5
0
-5
log2FPKM
dGE/amelo
GE
dGF
GF
Cytokine production
ZBTB20
NOD2
IL36B
APOA2
C3AR1
IL18R1
TNF
HERC5
TLR2
PLCG2
TLR3
C3
CSF1R
IRAK1
ISG15
SULF2
NOTCH1
TNFAIP3
SERPINB7
IRF7
MAF
GPNMB
IL6
RELB
DDX60
PELI1
IFIH1
ABCA1
CSF2
CCDC88B
RNF128
INPP5D
LTB
AFAP1L2
NDRG2
F2RL1
GPR56
CDH3
SAA1
8
6
4
2
0
-2
-4
-6
log2FPKM
dGE/amelo
GE
dGF
GF
Developmental process
PPP1R15A
TGM2
SSH3
SLC9A3R1
FLRT2
NCK2
TJP2
HMGA2
VAV2
SKIL
SMAD7
IL6
BAMBI
EPHB2
DDR1
JAG1
PDPN
ITGA2
NOTCH1
AKR1C2
ZNF385A
EFNB2
DUSP10
KRTAP2-3
GPR68
HIP1R
FKBP1B
MAF
JUP
F2RL1
IRF6
COL17A1
PTHLH
CDH3
TACSTD2
KRT17
KRT6A
LAMC2
LAMA3
LAMB3
ALDH1A2
KRTAP2-1
FGF13
UPK1A
CHODL
MAPT
RAP1GAP
BAI1
LCE2C
LCE1B
NKD1
C1orf68
COBL
WNT10A
VSIG1
FGD3
VAV1
CUX2
SEMA5B
CXCL10
SPP1
LST1
SRCIN1
RTN4R
CTSL2
S1PR5
FGD6
EPB49
SHANK3
L1CAM
MARVELD2
GPR56
CELSR1
PITX2
CYP1A1
EHF
RIPK4
PDGFB
TLR2
WNT7A
EFNA1
PRSS8
ST14
CASZ1
ZNF750
VAV3
PALMD
OVOL2
HPN
TNF
MMP12
BCL11B
FGD4
PTCH1
DACT2
EXPH5
ANKRD1
FOXC2
CSF1R
MAP2
KIF26B
SPRR2G
ICAM1
LRP4
EFNA5
FOXE1
DUOXA1
GPC4
FOXQ1
PLXNA2
IRS2
IVL
EDN1
NOG
HDAC9
NEO1
E2F7
SLC12A7
10
5
0
-5
log2FPKM
dGE/amelo
GE
dGF
GF

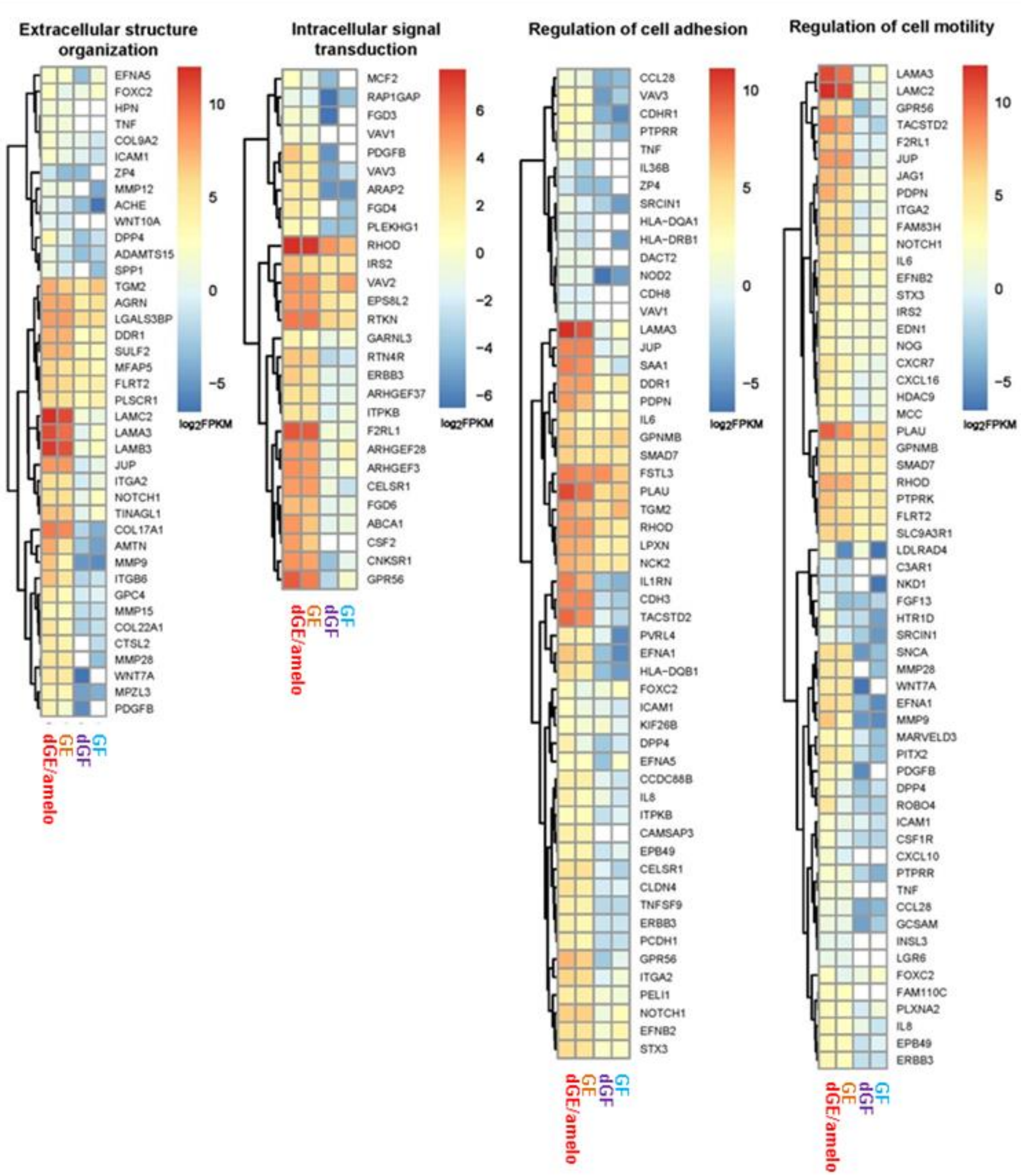


**Supplementary Figure S5. Gene Ontology enrichment analysis and the expression profiles of up-regulated genes in dGE/ameloblast induced by cytokines. (A)** Gene functional classification in 586 up-regulated genes was performed using the metascape tool. The X-axis in left and right side represented the significance level (scored as -$\log_{10}$($p$-value))

and number of genes that map to the corresponding GO terms, respectively. The Y-axis denoted the related functional GO terms (biological process in red, cellular component in green, and molecular function in sky blue). Among the 537 upregulated genes at dGE/amelo, 238 were significantly enriched in 49 GO terms (41 biological processes, 4 cellular components, and 4 molecular functions). **(B)** Heat map of up-regulated genes belonged to eight major GO categories: ***a*,** Cell surface; ***b*,** Cellular response to stimulus; ***c*,** Cytokine production; ***d*,** Development process; ***e*,** Extracellular structural organization; ***f*,** Intracellular signal transduction; ***g*,** Regulation of cell adhesion; ***h*,** Regulation of cell motility. $Log_2$-transformed FPKM values were used for creating the heat map.
